# D-Serine and Serine Racemase Are Associated with PSD-95 and Glutamatergic Synapse Stability

**DOI:** 10.3389/fncel.2016.00034

**Published:** 2016-02-25

**Authors:** Hong Lin, Ariel A. Jacobi, Stewart A. Anderson, David R. Lynch

**Affiliations:** ^1^Department of Pediatrics and Neurology, The Children's Hospital of PhiladelphiaPhiladelphia, PA, USA; ^2^University of Pennsylvania School of Arts and SciencesPhiladelphia, PA, USA; ^3^Department of Child and Adolescent Psychiatry and Behavioral Services, The Children's Hospital of PhiladelphiaPhiladelphia, PA, USA; ^4^University of Pennsylvania Perelman School of MedicinePhiladelphia, PA, USA

**Keywords:** D-serine, serine racemase, PSD-95, NMDA receptor, postsynaptic, glutamatergic synapse stability, cortical neurons, astrocytes

## Abstract

D-serine is an endogenous coagonist at the glycine site of synaptic NMDA receptors (NMDARs), synthesized by serine racemase (SR) through conversion of L-serine. It is crucial for synaptic plasticity and is implicated in schizophrenia. Our previous studies demonstrated specific loss of SR, D-serine-responsive synaptic NMDARs, and glutamatergic synapses in cortical neurons lacking α7 nicotinic acetylcholine receptors, which promotes glutamatergic synapse formation and maturation during development. We thus hypothesize that D-serine and SR (D-serine/SR) are associated with glutamatergic synaptic development. Using morphological and molecular studies in cortical neuronal cultures, we demonstrate that D-serine/SR are associated with PSD-95 and NMDARs in postsynaptic neurons and with glutamatergic synapse stability during synaptic development. Endogenous D-serine and SR colocalize with PSD-95, but not presynaptic vesicular glutamate transporter 1 (VGLUT1), in glutamatergic synapses of cultured cortical neurons. Low-density astrocytes in cortical neuronal cultures lack SR expression but contain enriched D-serine in large vesicle-like structures, suggesting possible synthesis of D-serine in postsynaptic neurons and storage in astrocytes. More interestingly, endogenous D-serine and SR colocalize with PSD-95 in the postsynaptic terminals of glutamatergic synapses during early and late synaptic development, implicating involvement of D-serine/SR in glutamatergic synaptic development. Exogenous application of D-serine enhances the interactions of SR with PSD-95 and NR1, and increases the number of VGLUT1- and PSD-95-positive glutamatergic synapses, suggesting that exogenous D-serine enhances postsynaptic SR/PSD-95 signaling and stabilizes glutamatergic synapses during cortical synaptic development. This is blocked by NMDAR antagonist 2-amino-5-phosphonopentanoic acid (AP5) and 7-chlorokynurenic acid (7-CK), a specific antagonist at the glycine site of NMDARs, demonstrating that D-serine effects are mediated through postsynaptic NMDARs. Conversely, exogenous application of glycine has no such effects, suggesting D-serine, rather than glycine, modulates postsynaptic events. Taken together, our findings demonstrate that D-serine/SR are associated with PSD-95 and NMDARs in postsynaptic neurons and with glutamatergic synapse stability during synaptic development, implicating D-serine/SR as regulators of cortical synaptic and circuit development.

## Introduction

NMDARs are glutamate-gated ionotropic channels that are crucial for many physiological processes including neurotransmission, synaptic plasticity, and learning and memory (Waxman and Lynch, 2005; Hardingham and Bading, 2010; Vyklicky et al., 2014). In addition to glutamate, NMDAR activation requires the binding of coagonist, D-serine or glycine, to the glycine site of NR1 subunit of NMDARs (Johnson and Ascher, 1987; Kleckner and Dingledine, 1988; Mothet et al., 2000, 2015; Labrie et al., 2008; DeVito et al., 2011; Papouin et al., 2012; Li and Wang, 2013; Rosenberg et al., 2013). In some paradigms, D-serine preferentially gates synaptic NMDARs and glycine preferentially gates extrasynaptic NMDARs (Papouin et al., 2012). D-serine is synthesized by serine racemase (SR) through conversion of L-serine and degraded by D-amino acid oxidase (DAAO) in various brain regions (Martineau et al., 2006; Wolosker, 2006, 2011; Labrie et al., 2009; Billard, 2012; Campanini et al., 2013; Van Horn et al., 2013). D-serine is now recognized as an important physiological modulator in many NMDAR-dependent processes and functions, including brain development, synaptic transmission and plasticity, learning and memory, and social interactions (Mothet et al., 2000, 2015; Yang et al., 2003; Kim et al., 2005a; Labrie et al., 2008; DeVito et al., 2011; Papouin et al., 2012; Li and Wang, 2013; Rosenberg et al., 2013).

Abnormal reduction of D-serine levels have been found in schizophrenia patients (Hashimoto et al., 2003, 2005; Yamada et al., 2005; Bendikov et al., 2007) and implicated in the pathogenesis of schizophrenia (Labrie et al., 2012). Targeted deletion or pathogenic perturbation of SR in mice reduces D-serine production and glutamatergic transmission in the forebrain and leads to schizophrenia-like behavior (Basu et al., 2009; Ma et al., 2013). SR null mutant mice, which have less than 10% of normal brain D-serine, have reduced dendritic spine density that can be partially rescued by chronic D-serine treatment (Balu and Coyle, 2012; Balu et al., 2012, 2014). Disruption of D-serine/SR has also been associated with schizophrenia during development. Neonatal disruption of SR and D-serine synthesis in mice leads to schizophrenia-like behavioral abnormalities in adulthood (Hagiwara et al., 2013). Previous studies have demonstrated that glutamatergic synapse formation and maturation are promoted by α7 nicotinic acetylcholine receptor during synaptic development (Lin et al., 2010; Lozada et al., 2012). Glutamatergic synapses and D-serine/SR are decreased in the forebrain of α7 nicotinic acetylcholine receptor knockout mice (Lin et al., 2014b). These changes resemble the major neurochemical deficits in schizophrenia (Lin et al., 2014a,b). We thus hypothesize that D-serine/SR may be involved in glutamatergic synaptic development, and that D-serine/SR deficiency may thereby disrupt cortical synaptic and circuit development, contributing to permanent deficits in schizophrenia.

The roles of D-serine/SR in neurons and astrocytes have been controversial. D-serine was characterized solely as a gliotransmitter in the initial studies showing D-serine and SR localized to astrocytes (Schell et al., 1997; Wolosker et al., 1999; Martineau et al., 2006; Panatier et al., 2006). Using more specific SR antibodies and SR-knockout mice as negative controls, SR is found preferentially expressed in excitatory and inhibitory neurons, and D-serine is predominantly produced and released by neurons in rodent and human brains (Kartvelishvily et al., 2006; Miya et al., 2008; Wolosker et al., 2008; Benneyworth et al., 2012; Ehmsen et al., 2013; Balu et al., 2014; Martineau et al., 2014; Mothet et al., 2015). SR interacts with DISC1 in astrocytes and pathogenic disruption of SR-DISC1 binding leads to depletion of D-serine levels and schizophrenia-like behavior in mice (Ma et al., 2013). SR interactions with stargazin and PSD-95 have been suggested to regulate NMDAR-AMPAR cross-talking in neurons (Ma et al., 2014), however the association of D-serine/SR with PSD-95 in postsynaptic neurons and their association with synapse stability remain unknown. In the current studies, we examined the expression and distribution of SR and D-serine and their association with glutamatergic synapses in primary cortical neuronal cultures containing low-density astrocytes from embryonic mice. Our findings reveal the association of D-serine/SR with PSD-95 and NMDARs in postsynaptic neurons and with glutamatergic synapse stability during cortical synaptic development based on the morphological and molecular evidence.

## Materials and methods

### Materials

Timed-pregnant C57BL/6 mice were purchased from Charles River Laboratories. Biochemicals included 2-amino-5-phosphonopentanoic acid (AP5), D-serine, L-serine, glycine and lithium (Sigma), 7-chlorokynurenic acid (7-CK) (Tocris Bioscience). Antibodies included α-NR1 (BD Pharmagen, mouse monoclonal, Millipore, mouse monoclonal), α-NR2A, α-NR2B (Alomone Labs, rabbit polyclonal), α-PSD-95 (BD Transduction Laboratories, mouse monoclonal; NeuroMab, mouse monoclonal), α-VGLUT1 (Synaptic Systems, polyclonal; mouse monoclonal), α-GAD65 (DSHB, mouse monoclonal), α-gephyrin (Synaptic systems, mouse monoclonal), α-D-serine, α-L-serine, α-Serine Racemase (Abcam, rabbit polyclonal), α-GFAP (Sigma, rabbit polyclonal; NeuroMab, mouse monoclonal), α-GLT1 (obtained from Dr. Michael B. Robinson, a courtesy of Dr. Jeffrey Rothstein, mouse monoclonal; NeuroMab, rabbit polyclonal), α-MAP2 (Abcam, chicken polyclonal), α-GABA (Sigma, rabbit polyclonal), α-VAMP2 (Synaptic systems, mouse monoclonal).

### Neuronal cultures and drug treatment

Primary cortical cultures from E17-19 C57BL/6 mice were prepared as described (Lin et al., 2010) in accordance with the protocol approved by The Children's Hospital of Philadelphia Institutional Animal Care and Use *Committee* (IACUC). Briefly, the cortex was dissected, gently minced, trypsinized (0.027%, 37°C; 7% CO_2_ for 20 min), and then washed with 1 × HBSS. Neurons were seeded to a density of 3 × 10^5^ viable cells in 35-mm culture dish with five 12-mm glass coverslips (low-density culture, 3 × 10^4^/cm^2^) or a density of 1.6 × 10^6^ viable cells in 60-mm culture dishes (high-density culture, 8 × 10^4^/cm^2^). The culture dishes were coated with poly-D-Lysine (100 μg/ml) prior to seeding neurons. Neurons were maintained at 37°C with 5% CO_2_ in Neurobasal medium with B27 supplement. Cortical cultures contain 5–10% of glia cells and 90–95% cortical neurons. At 17–19 (high-density cultures, 8 × 10^4^/cm^2^) or 23–25 (low-density cultures, 3 × 10^4^/cm^2^) *days in vitro* (DIV), cultures were subject to drug treatment for 24 h, western blotting analysis, co-immunoprecipitation and immunocytochemistry. For drug treatment, the cortical cultures were treated with vehicle, D-serine (50 μM), D-serine (50 μM) + AP5 (50 μM), D-serine (50 μM) + 7-CK (50 μM), glycine (50 μM) + lithium (100 μM) for 24 h.

For cell lysate preparation, cultures were lysed in lysis buffer (150 mM NaCl, 1 mM EDTA, 100 mM Tris-HCl, 1% Triton X-100, 1% sodium deoxycholate and 1% SDS, pH 7.4, supplemented the day of use with 1:500 EDTA-free protease inhibitor cocktail III (Calbiochem) for 1 h at 4°C and collected. Debris was cleared by centrifugation at 16,100 × g for 20 min at 4°C. Supernatants were stored at −80°C until use.

### Co-immunoprecipitation and western blotting analysis

Co-immunoprecipitation and Western blotting was performed as described previously (Zhai et al., 2005). Protein content of cortical lysates was determined using BCA Protein Assay (Thermo Scientific). Equal amounts of total protein lysates (250 μg) were first added 2 μg primary antibody (α-SR, α-NR1, or α-PSD-95) or normal IgG and incubated at 4°C for 2h. Immunocomplexes were then precipitated with protein A or protein G-agarose beads shaking overnight at 4°C, washed twice in lysis buffer, eluted by boiling in SDS-PAGE sample buffer, and subjected to Western blot analysis. Equal volumes of eluted buffers for co-immunoprecipitation assay or equal amounts of total protein (15 μg cell lysate) for protein input analysis were subjected to 4–12% NuPAGE Gel for electrophoresis and transferred to nitrocellulose membranes. Membranes were blocked with 3% nonfat milk and incubated with primary antibody overnight at 4°C. Blots were then incubated with appropriate horseradish peroxidase, HRP-conjugated secondary antibodies (Cell Signaling) for 2 h at room temperature and then washed; Reaction bands were visualized using a luminol-enhanced chemiluminescence (ECL) HPR substrate (Thermo Scientific). Each blot was then incubated with stripping buffer (2% SDS, 50 mM Tris, pH 6.8, and 100 mM β-mercaptoethanol) for 45 min at room temperature to remove the signals and reprobed for other proteins. For quantification analysis, reaction product levels were quantified by scanning densitometry and the ratio of co-precipitated protein was normalized by input levels from 3 different cultures and experiments using NIH Image J software.

### Immunocytochemistry and fluorescence imaging

Primary cultured cortical neurons were fixed for 20 min at 4°C with 4% paraformaldehyde in phosphate-buffered saline (PBS) (pH 7.4), and then subjected to the immunostaining procedure. For immunostaining procedure, after blocking with 5% normal goat serum and 1% bovine serum albumin in combination with 0.3% (vol/vol) Triton X-100 in PBS at room temperature for 1 h, the coverslips or slides were incubated with primary antibodies at 4°C overnight and then secondary antibodies conjugated to Alexa fluor 488 or 568 or 647 (Invitrogen) at room temperature for 60–90 min. Following several washes with PBS, cells or slides were mounted with Vectashield with DAPI (Vector Laboratories). Fluorescence images were obtained with Olympus FluoView and Leica TCS SP8 laser scanning confocal microscope. For quantification analysis of glutamatergic synapses in cortical cultures, neurons were stained for glutamatergic synaptic markers (VGLUT1 and PSD-95). The confocal images were acquired under 40x objectives with zoom x6 from the dendrites of 5 neurons and 3 different cultures for quantification of VGLUT1-positive puncta on the dendrites of cortical neurons. NIH Image J software and the thresholded images were used to quantify the number of glutamatergic synapses on the primary and secondary dendrites of cortical neurons. Since nearly 100% of VGLUT1-positive puncta were colocalized with or adjacent to one or more PSD-95-positive puncta in the dendrites of cortical neurons, the number of VGLUT1-positive puncta reasonably represents the number of glutamatergic synapses on the dendrites (Lin et al., 2014b).

### Statistical analysis

Data was shown as the mean ± S.E.M. Experiments were analyzed using Student's *t*-test to compare two conditions or ANOVA followed by planned comparisons of multiple conditions. Significance was set at *P* < 0.05.

## Results

### Serine racemase (SR) colocalizes with PSD-95, but not presynaptic VGLUT1, in glutamatergic synapses of cortical glutamatergic and gabaergic neurons

**Figure 1:**
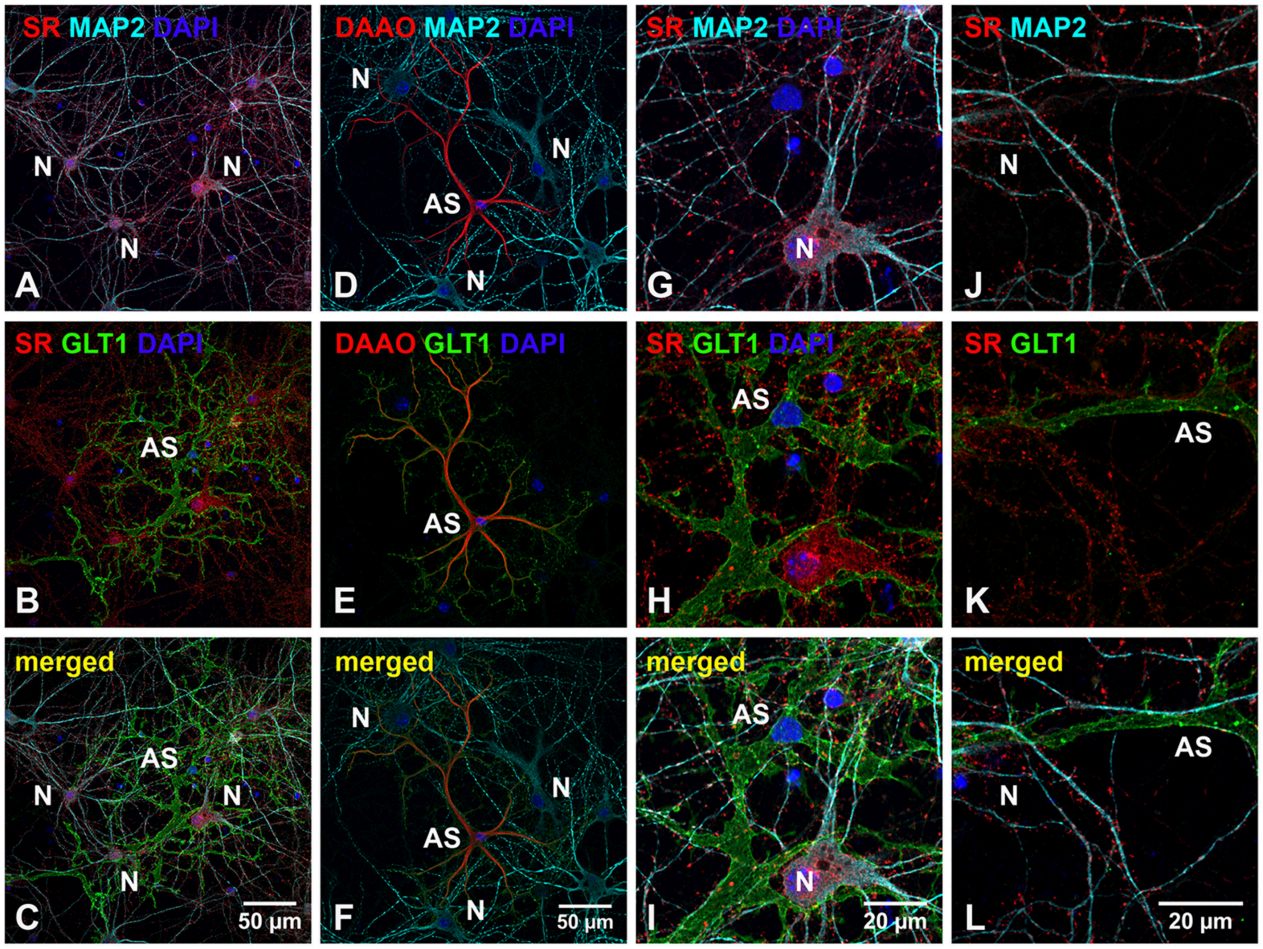
**SR is abundant in cortical neurons but absent in low-density astrocytes in primary cortical neuronal cultures. (A–F)** Confocal images of SR or DAAO immunofluorescence (red), GLT1 immunofluorescence (green), MAP2 immunofluorescence (cyan) and DAPI staining (blue) in primary cortical neuronal cultures with low-density astrocytes showing that SR appears abundantly in the MAP2-positive cortical neurons (N) **(A)** but absent in GLT1-positive astrocytes (AS) **(B)**. D-serine degrading enzyme DAAO is abundantly present in the GLT1-positive astrocytes (AS) **(E)** but absent in MAP2-positive cortical neurons (N) **(D)**. **(G–L)** Higher magnification of confocal images showing that SR appears abundant in the synaptic-like structures on MAP2-positive dendrites of cortical neurons (N), but absent in GLT1-positive astrocytes (AS). Scale bars as indicated.

SR is an endogenous biosynthetic enzyme that converts L-seine to D-serine, and DAAO is an endogenous D-amino acid oxidase that degrades D-serine in the nervous system. To explore the possible role of D-serine in cortical synaptic development, we first examined the expression and distribution of SR and DAAO in primary cortical neuronal cultures at 28 *days in vitro* (DIV) by immunocytochemical studies using antibodies to SR, DAAO, astrocytic marker (glutamate transporter 1, GLT1) and neuronal markers (Microtubule-associated protein 2, MAP2). Immunostaining with astrocytic and neuronal marker antibodies indicates that cortical neuronal cultures contain 5–10% glial cells and 90–95% cortical neurons. Triple immunostaining with α-SR or α-DAAO and α-MAP2 and α-GLT1 show that SR is abundantly distributed in the soma, nucleus and dendrites of MAP2-positive cortical neurons (N) (Figure 1A), but absent in the soma and astrocytic terminals of GLT1-positive astrocytes (AS) (Figure 1B) in cortical neuronal cultures (Figure 1C). In contrast, DAAO is abundantly distributed in the soma of GLT1-positive astrocytes (AS) (Figure 1E), but absent in MAP2-positive neurons (N) (Figure 1D) in cultures (Figure 1F), suggesting possible synthesis of D-serine in neurons and degradation in astrocytes. High magnification confocal images further show that the majority of SR appears as puncta in the synaptic-like structures (Figures 1H,K) on the MAP2-positive dendrites (Figures 1G,J). Noticeably, some SR-positive puncta on the MAP2-positive dendrites are close to or overlap with GLT1-positive astrocytic membranes (Figures 1I–L).

To define the nature of SR distribution in the synaptic-like structures on the dendrites, we examined whether SR is located in glutamatergic or GABAergic synapses using antibodies to glutamatergic and GABAergic presynaptic and postsynaptic markers. We first characterized cortical glutamatergic and GABAergic neurons in cultures using the GABAergic neuronal marker GABA as well as glutamatergic markers PSD-95 and VGLUT1. In GABA-positive cortical GABAergic interneurons, GABA is distributed in the soma, dendrites as well as axonal and presynaptic terminals surrounding glutamatergic neurons (Supplemental Figures 1A–C), and PSD-95 is localized in the shaft-like synapses on the somatic and dendritic membrane (Supplemental Figures 1A–F). In cortical glutamatergic neurons surrounded by GABA-positive synaptic terminals, PSD-95 is distributed in the soma and in the spine-like synapses on the dendrites (Supplemental Figures 1G–I). Co-immunostaining with PSD-95 and VGLUT1 antibodies further confirms PSD-95- and VGLUT1-positive shaft-like synapses on cortical GABAergic interneurons (Supplemental Figures 1J–L) as well as dendritic spine-like synapses on cortical glutamatergic neurons (Supplemental Figures 1M–O).

**Figure 2:**
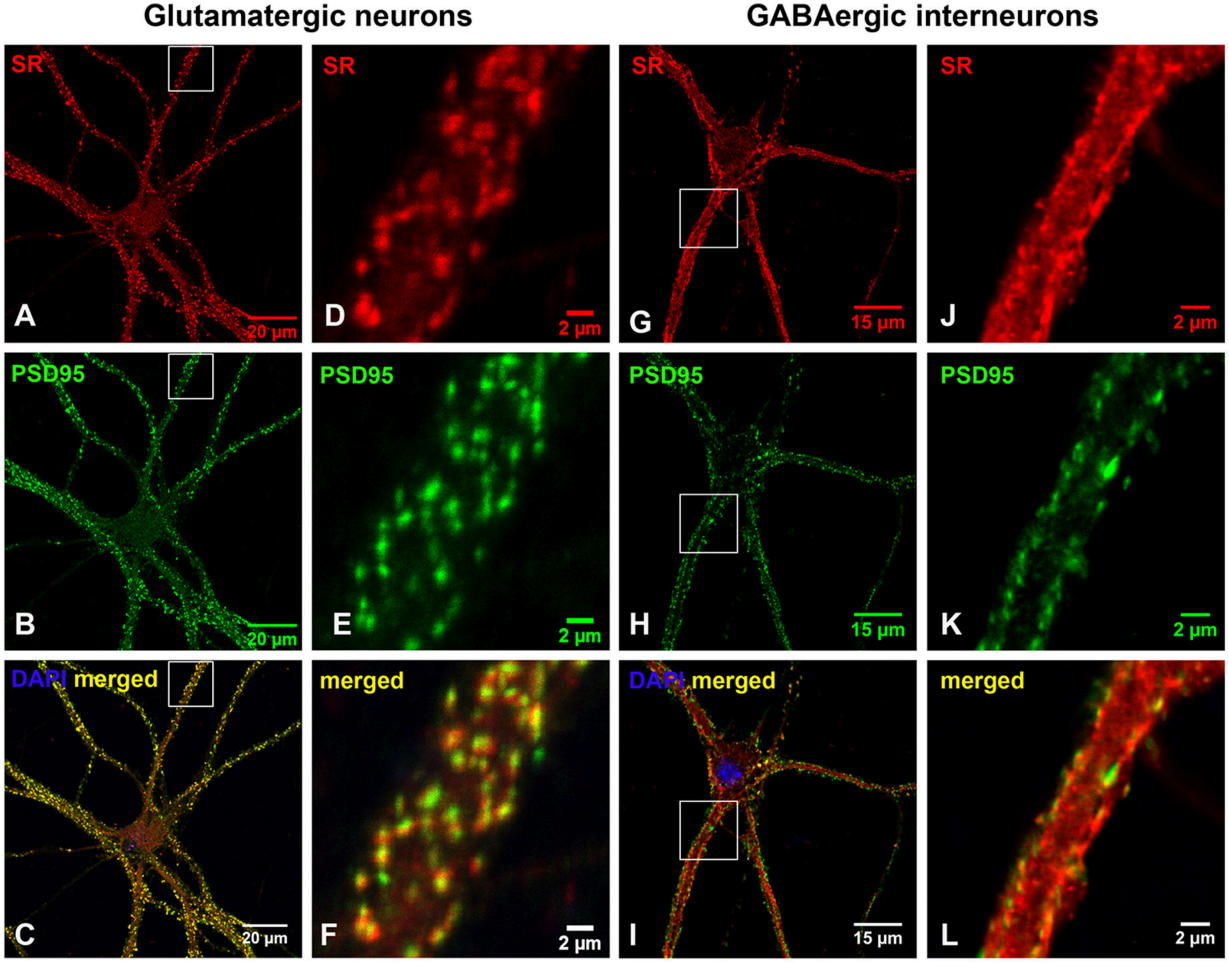
**SR is a postsynaptic protein that colocalizes with PSD-95 in the glutamatergic synapses on cortical glutamatergic and GABAergic neurons**. Confocal images of SR immunofluorescence (red) and PSD-95 immunofluorescence (green) showing that SR appears as puncta in the soma, nucleus and dendrites of cortical neurons **(A,D,G,J)** in primary cortical neuronal cultures at 28 DIV. SR colocalizes with PSD-95-positive glutamatergic postsynaptic terminals **(B,E,H,K)** on the dendrites of cortical glutamatergic **(C,F)** and GABAergic **(I,L)** neurons as characterized by PSD-95 immunofluorescence shown in Supplemental Figure 1. Scale bars as indicated.

**Figure 3:**
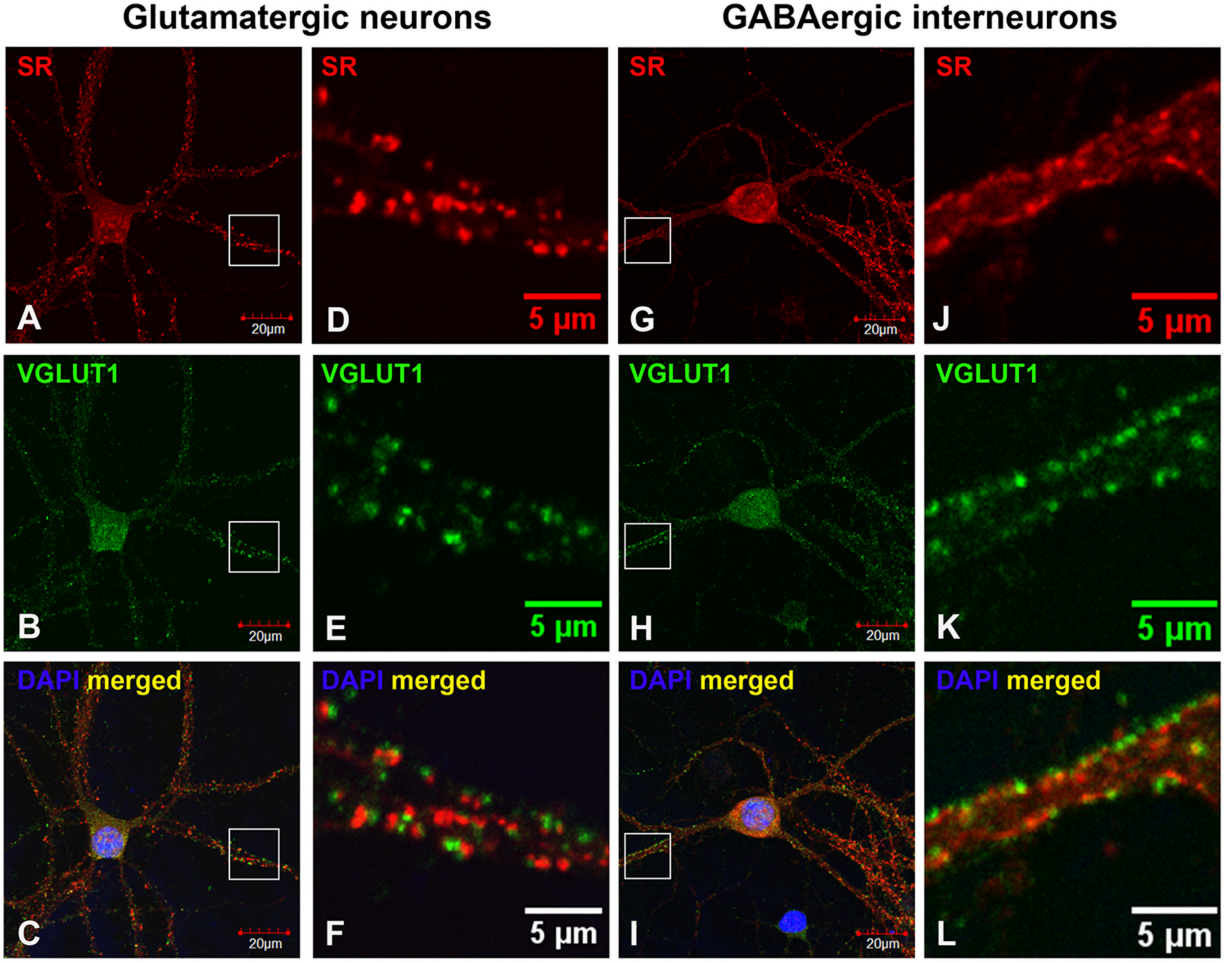
**SR is absent in glutamatergic presynaptic terminals on cortical glutamatergic and GABAergic neurons**. Confocal images of SR immunofluorescence (red) and VGLUT1 immunofluorescence (green) showing that SR appears as puncta in the soma, nucleus and dendrites of cortical neurons **(A,D,G,J)**. SR does not colocalize with VGLUT1-positive glutamatergic presynaptic terminals **(B,E,H, K)** on cortical glutamatergic **(C,F)** and GABAergic **(I,L)** neurons as characterized by VGLUT1 and PSD-95 immunofluorescence shown in Supplemental Figure 1. Scale bars as indicated.

Based on co-immunostaining with antibodies to glutamatergic presynaptic and postsynaptic markers (VGLUT1 and PSD-95), SR colocalizes with PSD-95-positive glutamatergic postsynaptic terminals on the dendrites of cortical glutamatergic (Figures 2A–F) and GABAergic neurons (Figures 2G–L), but does not colocalize with VGLUT1-positive glutamatergic presynaptic terminals on the dendrites of cortical glutamatergic (Figures 3A–F) and GABAergic neurons (Figures 3G–L). In addition, co-immunostaining with antibodies to GABAergic presynaptic and postsynaptic markers (GAD65 and gephyrin) shows that SR does not colocalize with GAD65-positve GABAergic presynaptic (Supplemental Figures 2A–F) or gephyrin-positive GABAergic postsynaptic terminals (Supplemental Figures 2G–L) on cortical glutamatergic neurons (Supplemental Figures 2A–C,G–I) and GABAergic neurons (Supplemental Figures 2D–F,J–L). Taken together, these results indicate that SR is a postsynaptic protein that colocalizes with PSD-95 in glutamatergic synapses of cortical neurons.

### Endogenous D-serine colocalizes with PSD-95-positive glutamatergic postsynaptic terminals on cortical glutamatergic and gabaergic neurons

**Figure 4:**
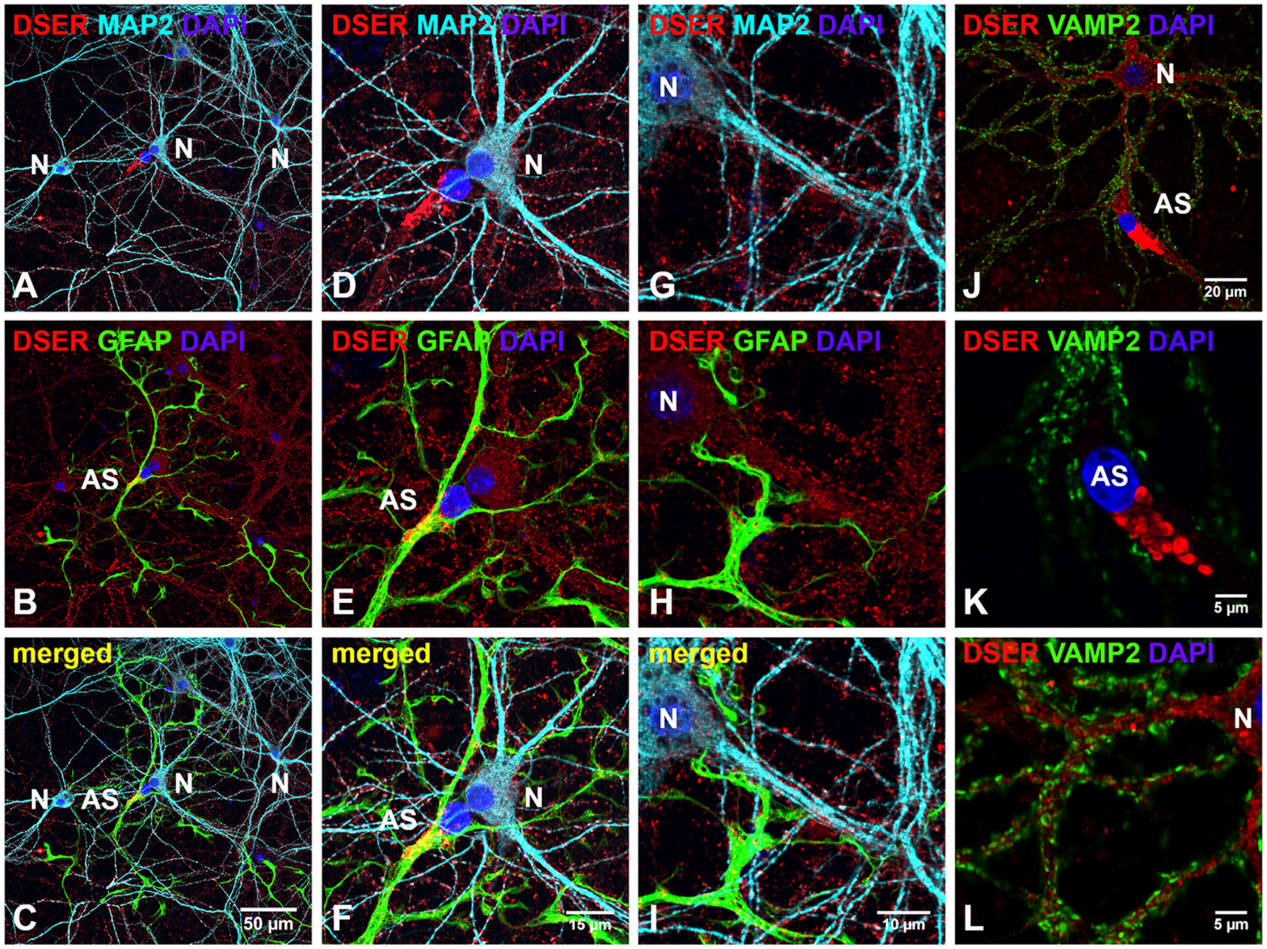
**D-serine is abundant in cortical neurons and enriched in low-density astrocytes in primary cortical neuronal cultures. (A–I)** Confocal images of D-serine immunofluorescence (red), GFAP immunofluorescence (green), MAP2 immunofluorescence (cyan), and DAPI nucleus staining (blue) showing that D-serine appears abundantly in the soma and dendrites of MAP2-positive cortical neurons (N) **(A)** and in the soma of GFAP-positive astrocytes (AS) **(B)**. Higher magnification of confocal images **(D–I)** showing that D-serine appears abundant in the synaptic-like structures on MAP2-positive dendrites **(D,G)** and enriched in large vesicle-like structures (1–3 μm) in the soma of GFAP-positive astrocytes **(E,H)**. **(J–L)** Confocal images of D-serine immunofluorescence (red), VAMP2 immunofluorescence (green), and DAPI staining (blue) showing that D-serine appears abundant in the soma and dendrites of cortical neurons surrounded by VAMP2-positive presynaptic terminals (N) and enriched in the soma of VMAP2-negative astrocytes (AS) **(J)**. Higher magnification of confocal image confirms that D-serine appears in large vesicle-like structures (1–3 μm) in the soma of VMAP2-negative astrocytes (AS) **(K)** and in the dendrites of cortical neurons surrounded by VAMP2-positive presynaptic terminals **(L)**. Scale bars as indicated.

The distribution of D-serine was directly examined in cortical cultures using triple immunostaining with α-D-serine antibody along with antibodies to neuronal (α-MAP2) and astrocytic markers (α-GFAP). To determine the specificity of α-D-serine antibody, we first compared the immunoreactivities of α-D-serine and α-L-serine antibodies, D-serine appears abundant as puncta in the soma and dendrites of MAP2-positive cortical neurons and in the soma of astrocytes (Supplemental Figures 3A–D), whereas L-serine is diffusely distributed in GFAP-positive astrocytes and very low in cortical neurons (Supplemental Figures 3E–H), suggesting the specificity of D-serine immunoreactivity and matching the findings on astrocytic supply of L-serine (Ehmsen et al., 2013). Furthermore, D-serine is abundant in the soma and in the synaptic-like structures on the MAP2-positive (Figures 4A,D,G) dendrites of cortical neurons (N) in cortical cultures (Figures 4C,F,I), whereas D-serine appears enriched as large puncta in the soma of GFAP-positive (Figures 4B,E,H) astrocytes (AS). Co-immunostaining with synaptic vesicle membrane associated protein 2 (VAMP2) antibody shows that D-serine appears enriched in large vesicle-like structures (1–3 μm) which are VAMP2-negative in the soma of astrocytes (AS) (Figures 4J–L), and that D-serine is abundant in the dendrites but absent in the VAMP2-positve presynaptic terminals on the dendrites of cortical neurons (Figures 4J,L).

**Figure 5:**
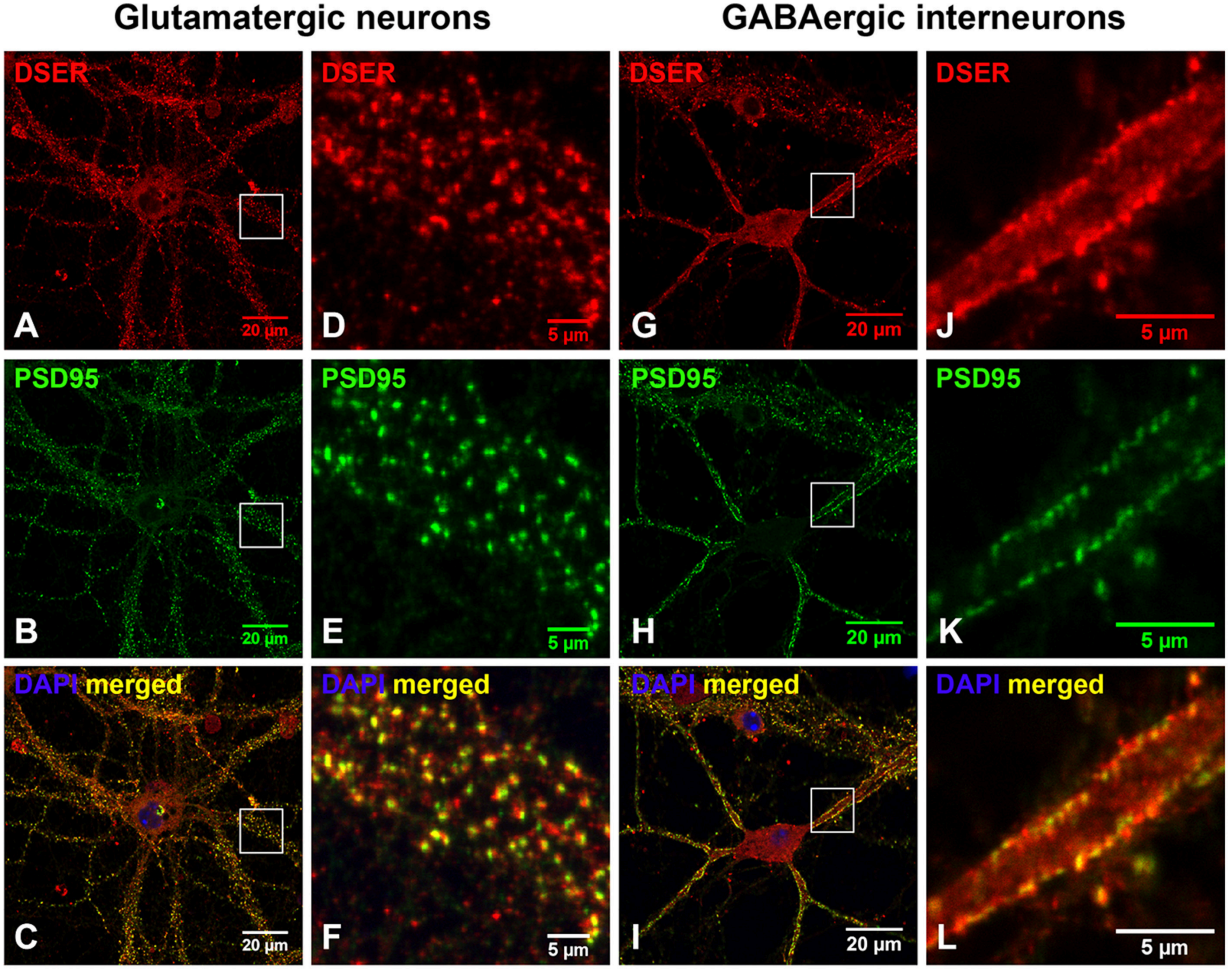
**D-serine colocalizes with PSD-95-positive postsynaptic terminals on cortical glutamatergic and GABAergic neurons. (A–F)** Confocal images of D-serine immunofluorescence (red), PSD-95 immunofluorescence (green), and DAPI nucleus staining (blue) showing that D-serine **(A,D)** colocalizes with PSD-95 **(B,E)** in the spine-like synapses on the dendrites of cortical glutamatergic neurons in primary cortical neuronal cultures at 28 DIV **(C,F)**. **(G–L)** Confocal images showing that D-serine **(G,J)** colocalizes with PSD-95 **(H,K)** in the shaft-like synapses on the dendrites of cortical GABAergic neurons **(I,L)** as characterized by PSD-95 immunofluorescence shown in Supplemental Figure 1. Scale bars as indicated.

**Figure 6:**
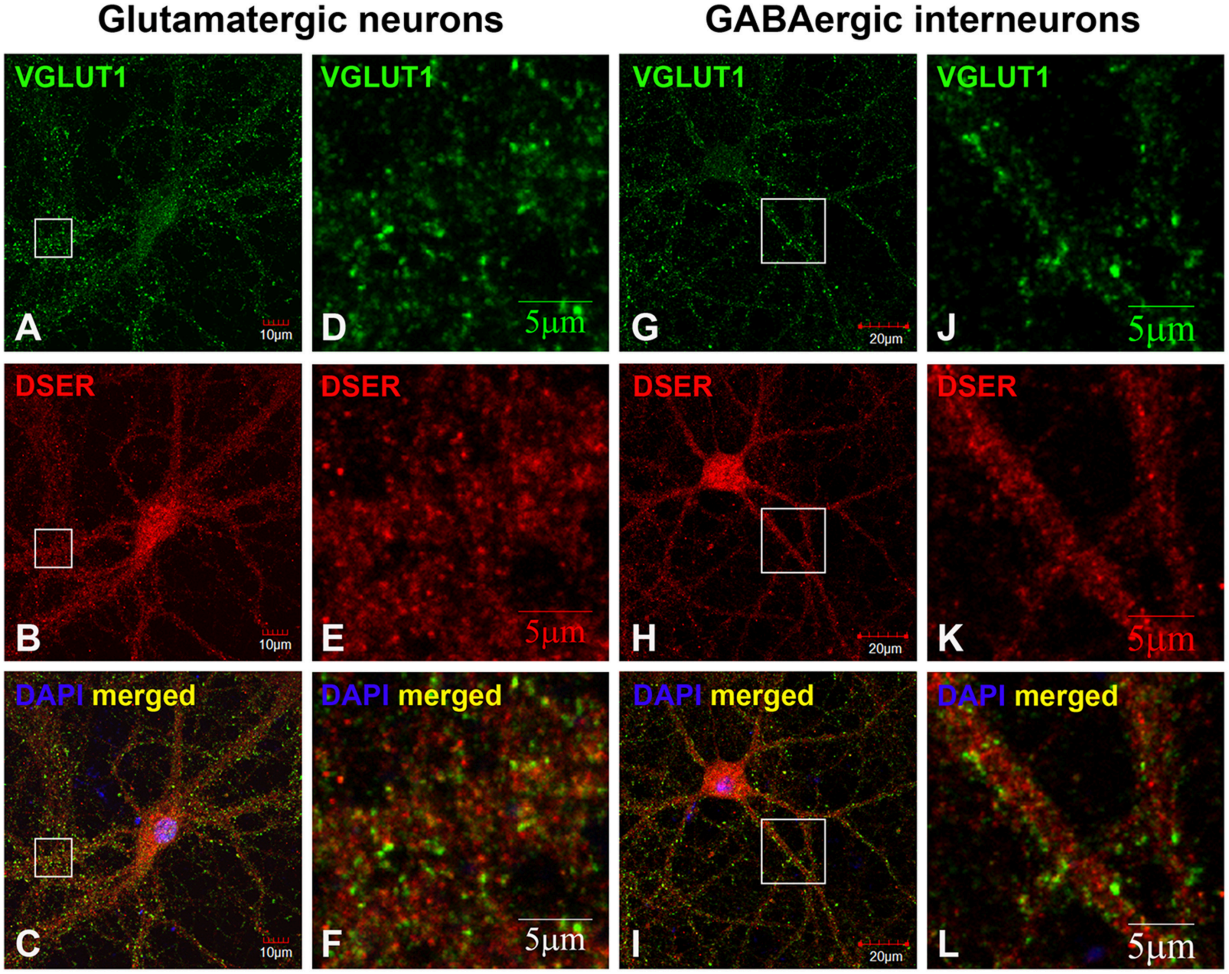
**D-serine is absent in glutamatergic presynaptic terminals on cortical glutamatergic and GABAergic neurons**. Confocal images of D-serine **(B,E,H,K)** red immunofluorescence (red) and VGLUT1 **(A,D,G,J)** immunofluorescence (green) showing that D-serine does not colocalize with VGLUT1-positive glutamatergic presynaptic terminals on cortical glutamatergic **(C,F)** and GABAergic **(I,L)** neurons as characterized by VGLUT1 and PSD-95 immunofluorescence shown in Supplemental Figure 1. Scale bars as indicated.

We further defined the localization of D-serine within glutamatergic presynaptic and postsynaptic terminals of cortical neurons. Co-immunostaining with α-D-serine and α-PSD-95 antibodies shows that D-serine appears in the synaptic-like structures that colocalize with PSD-95 on the dendrites of cortical glutamatergic (Figures 5A–C) and GABAergic neurons (Figures 5G–I). High magnification of confocal images confirms that D-serine colocalizes with PSD-95-positive postsynaptic terminals in the spine-like synapses on cortical glutamatergic neurons (Figures 5D–F) and in the shaft-like synapses on cortical GABAergic interneurons (Figures 5J–L), suggesting the localization of D-serine in postsynaptic neurons. Moreover, co-immunostaining with antibodies to D-serine and the glutamatergic presynaptic terminal marker α-VGLUT1 shows that D-serine is adjacent to, but does not colocalize with, VGLUT-positive glutamatergic presynaptic terminals on cortical glutamatergic (Figures 6A–F) and GABAergic neurons (Figures 6G–L). Taken together, our findings demonstrate D-serine association with PSD-95 in postsynaptic neurons and possible storage of D-serine in astrocytes.

### Endogenous SR and D-serine colocalize with PSD-95 in the postsynaptic terminals of glutamatergic synapses of cortical neurons during early synaptic development

**Figure 7:**
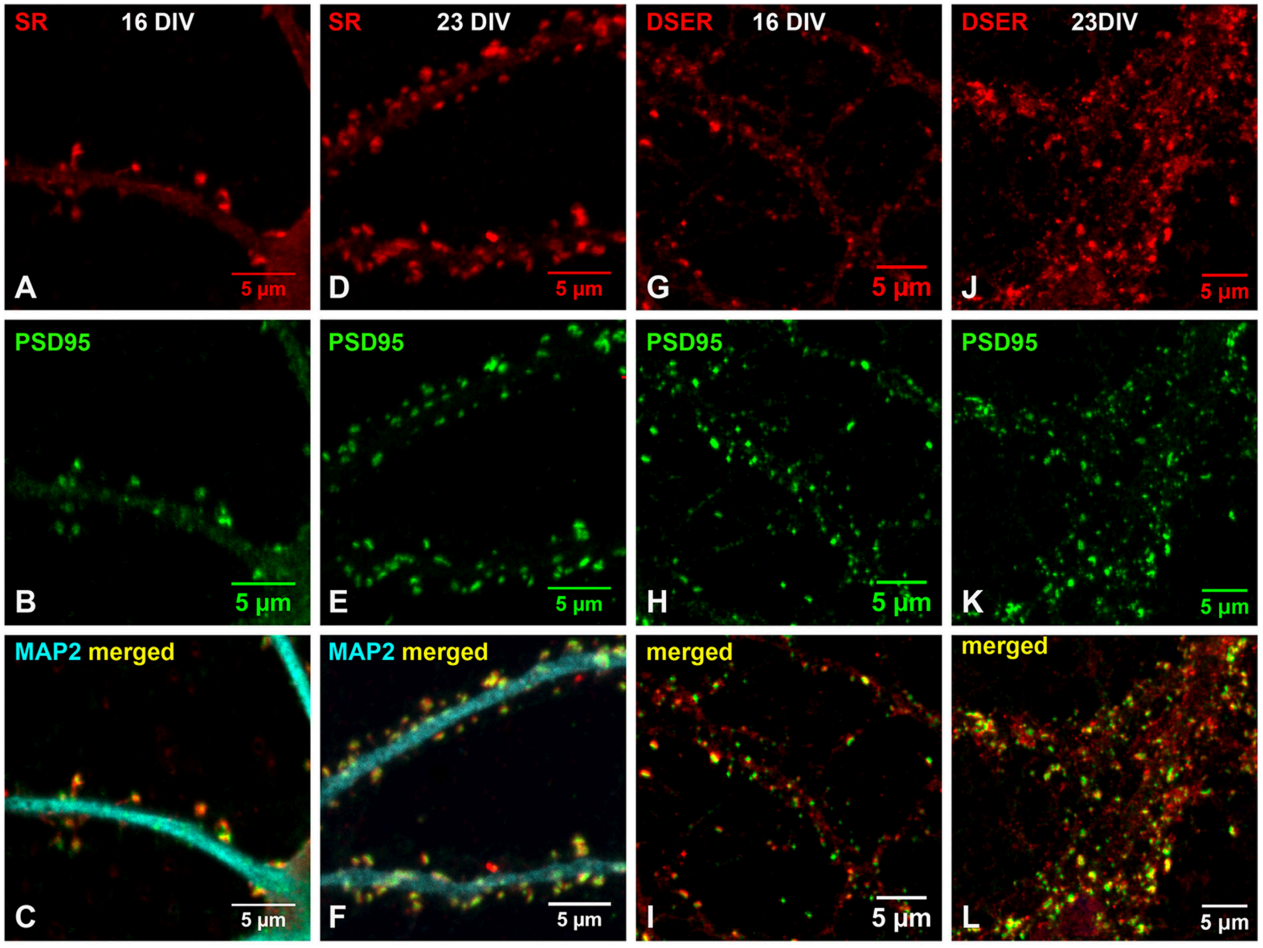
**Endogenous SR and D-serine colocalize with PSD-95 in the postsynaptic terminals of glutamatergic synapses during early synaptic development. (A–F)** Confocal images of SR immunofluorescence (red), PSD-95 immunofluorescence (green), and MAP2 immunofluorescence (cyan) showing colocalization of SR **(A,D)** with PSD-95 **(B,E)** in the spine-like synapses on the MAP2-positive dendrites **(C,F)** of cortical neurons at 16DIV **(A–C)** and 23 DIV **(D–F)**. **(G–L)** Confocal images of D-serine immunofluorescence (red) and PSD-95 immunofluorescence (green) showing colocalization of D-serine **(G,J)** with PSD-95 **(H,K)** in the spine-like synapses on the dendrites **(I,L)** of cortical neurons at 16DIV **(G–I)** and 23 DIV **(J–L)**. Scale bars as indicated.

Previous findings suggest that D-serine/SR may be involved in glutamatergic synaptic development (Balu et al., 2013, 2014; Ma et al., 2013; Lin et al., 2014b). To test this, we further examined the association of D-serine/SR with PSD-95 in early cortical cultures (16 DIV and 23 DIV). Co-immunostaining with α-SR and α-PSD-95 antibodies shows that, in cortical neuronal cultures at 16 DIV, SR (Figure 7A) is located in the postsynaptic terminals of spine-like synapses and colocalizes with PSD-95 (Figure 7B) on the MAP2-positive dendrites (Figure 7C) of cortical neurons. At 23 DIV, the number of glutamatergic synapses increases on the MAP2-positive dendrites of cortical neurons, and SR colocalizes with PSD-95 in the postsynaptic terminals of glutamatergic synapses (Figures 7D–F). Co-immunostaining with α-D-serine and α-PSD-95 antibodies shows similar co-localization of D-serine (Figures 7G,J) and PSD-95 (Figures 7H,K) in the postsynaptic terminals of glutamatergic synapses (Figures 7I,L) in early cortical cultures at 16 DIV and 23 DIV, suggesting that D-serine/SR may be involved in PSD-95 signaling and glutamatergic synaptic development.

### Exogenous D-serine, but not glycine, enhances the *In vivo* interactions of SR with PSD-95 and NMDARs postsynaptically

**Figure 8:**
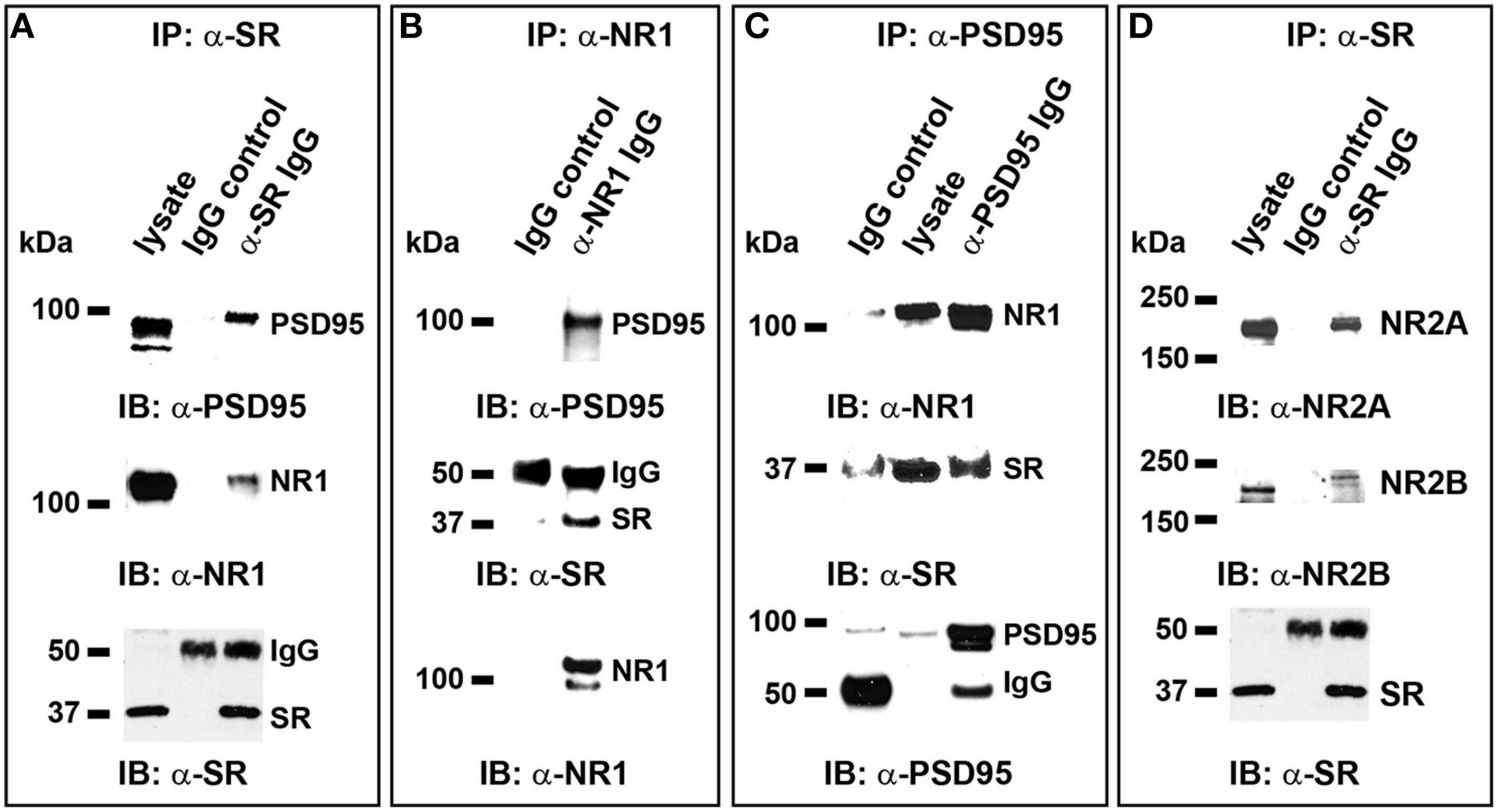
***In vivo* interactions of endogenous SR with PSD-95 and NMDARs postsynaptically in cortical neurons. (A)** In normal cortical neurons, co-immunoprecipitation (IP) of endogenous PSD-95 and NR1 with SR antibody in cortical neuronal lysates was observed, but co-precipitation with normal IgG was not; **(B)**, Co-IP of endogenous PSD-95 and SR with NR1 antibody in cortical neuronal lysates was observed, but co-precipitation with normal IgG was not; **(C)**. Co-IP of NR1 and SR with PSD-95 antibody was observed, but co-precipitation with normal IgG was not; **(D)**. Co-IP of NR2A and a lesser amount of NR2B with SR antibody in cortical neuronal lysates was observed, but co-precipitation with normal IgG was not.

To explore the possible involvement of D-serine/SR in postsynaptic PSD-95 signaling, we examined the effects of exogenous D-serine application on PSD-95 interactions with SR and NMDARs in cortical cultures. We first confirmed the *in vivo* interactions of endogenous SR with PSD-95 in cortical neurons using co-immunoprecipitation. In cortical neuronal lysates, α-SR antibody co-precipitates PSD-95 as well as NR1 (Figure 8A). Similarly, α-NR1 antibody co-precipitates SR and PSD-95 in cortical neuronal lysates (Figure 8B), and incubation of cortical neuronal lysates with α-PSD-95 antibody co-precipitates NR1 and SR (Figure 8C). Furthermore, α-SR antibody co-precipitates NR2A and a lesser amount of NR2B in cortical neuronal lysates (Figure 8D). These findings are not observed with control IgG in these assays (Figures 8A–D). This identifies the *in vivo* interactions of SR with PSD-95, NR1 and NR2 in a postsynaptic protein complex.

**Figure 9:**
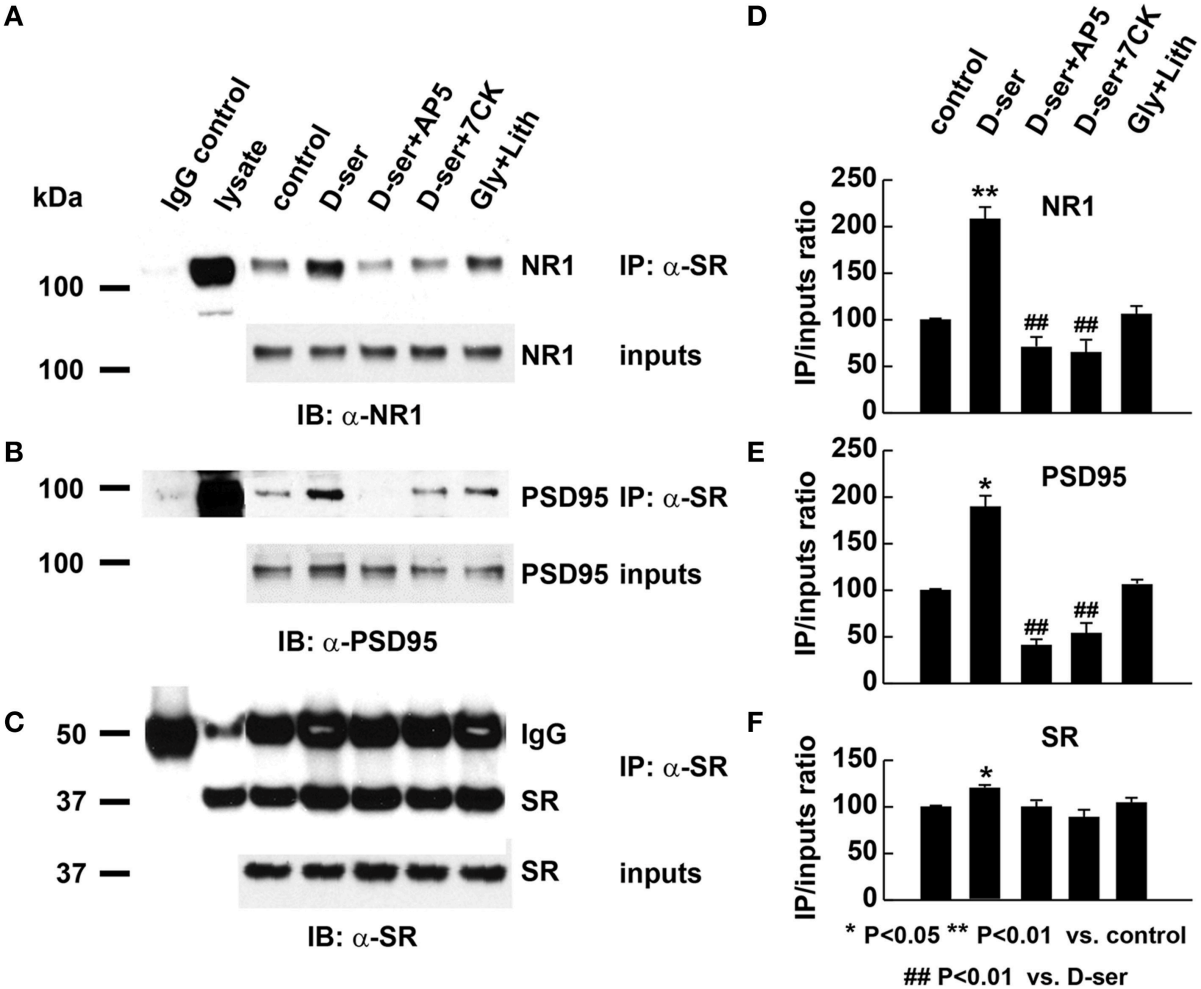
**Exogenous D-serine, but not glycine, enhances the *in vivo* interactions of endogenous SR with PSD-95 and NMDARs postsynaptically. (A–C)** Co-immunoprecipitation of endogenous NR1 and PSD-95 with SR antibody in cortical neuronal lysates from cultures treated with vehicle, D-serine (50 μM), D-serine (50 μM) + AP5 (50 μM), D-serine (50 μM) + 7-CK (50 μM), glycine (50 μM) + lithium (100 μM) for 24 h. Upper panel: Co-immunoprecipitation assays of cortical lysates (250 μg each lane); Lower panel: Western blot analysis of input levels in cortical lysates (15 μg each lane). Graphs **(D–F)** show quantification analysis of the co-precipitated NR1, PSD-95, and SR with α-SR antibody in the cortical lysates from 3 different cultures and experiments. The co-precipitated products were normalized to inputs levels in cortical lysates.

To explore the possible role of D-serine in postsynaptic functions, we examined the effects of exogenous application of D-serine on the *in vivo* interactions of SR with PSD-95 and NMDARs using co-immunoprecipitation assays with α-SR antibody. Exogenous application of D-serine markedly increases the amount of PSD-95 and NR1 co-immunoprecipitated by α-SR antibody from the same amount of cortical neuronal lysate, even though the levels of PSD-95, NR1 and SR inputs remain similar between the control and treatment groups (Figures 9A–C). The ratios of co-precipitated NR1 and PSD-95 normalized to input controls are significantly increased in D-serine-treated cortical lysates (Figures 9D–F). The results suggest that exogenous D-serine enhances the *in vivo* interactions of SR with NR1 and PSD-95. This enhancement is blocked by co-application of AP5, an NMDAR competitive antagonist, as well as with 7-chlorokynurenic acid (7-CK), a specific NMDAR competitive antagonist at the glycine site of NMDARs (Figures 9A–F), demonstrating that D-serine modulates SR/PSD-95 interactions through NMDAR activation. In contrast, exogenous application of glycine along with glycine transporter inhibitor lithium, does not have the same effect on SR interactions with NR1 and PSD-95 as exogenous D-serine (Figures 9A–F), suggesting that D-serine, rather than glycine, modulates postsynaptic SR interactions with PSD-95 and NMDARs.

### Exogenous D-serine, but not glycine, stabilizes cortical glutamatergic synapses

**Figure 10:**
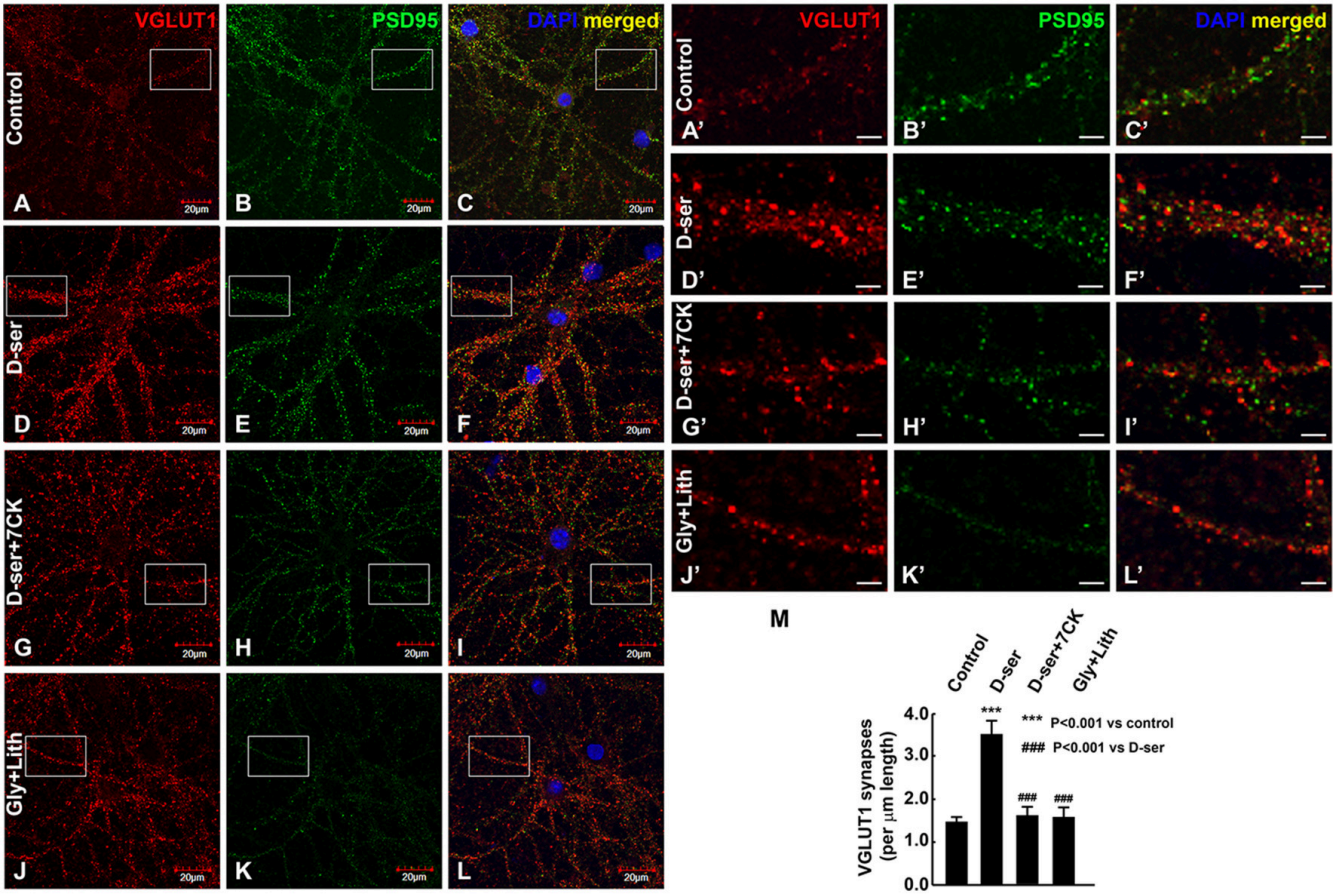
**Exogenous D-serine, but not glycine, increases the number of glutamatergic synapses in cortical cultures**. Confocal images **(A–L)** of α-VGLUT1 **(A,D,G,J)** immunofluorescence (red) and α-PSD-95 **(B,E,H,K)** immunofluorescence (green) shows that VGLUT1- and PSD-95-positive glutamatergic synapses surrounding cortical neurons are markedly increased by 50 μM exogenous D-serine **(D–F)**, but not 50 μM glycine + 100 μM lithium **(J–L)** compared with control cortical neurons **(A–C)**. This increase can be blocked by co-application of 50 μM 7-CK with 50 μM D-serine **(G–I)**. Higher magnification of confocal images **(A'–L')** showing VGLUT1- and PSD-95-positive glutamatergic synapses on the dendrites of cortical neurons in control **(A'–C')**, D-serine **(D'–F')**, D-serine+7-CK **(G'–I')**, glycine+lithium **(J'–L')** groups. Graph **(M)** shows quantification of the number of VGLUT1-positive glutamatergic synapses on the dendrites of cortical neurons from 3 different cultures and experiments. Scale bars as indicated.

We then examined the effects of exogenous D-serine on glutamatergic synapses in cortical cultures (23-25DIV) by immunocytochemical studies with antibodies to glutamatergic synaptic markers (α-VGLUT1 and α-PSD-95). Exogenous application of D-serine for 24 h dramatically increases the number of VGLUT1- and PSD-95-positive synapses in cortical cultures (Figures 10D–F) compared with control cultures (Figures 10A–C) with a more pronounced effect on presynaptic terminals. This suggests that D-serine may increase the number of glutamatergic synapses through synapse stabilization. The increase in synapse number is blocked by the NMDAR antagonist 7-CK (Figures 10G–I), whereas exogenous application of glycine along with the glycine transporter inhibitor lithium does not increase the number of glutamatergic synapses (Figures 10J–L). Higher magnification of confocal images further show that D-serine, but not glycine, increases the number of VGLUT-1- and PSD-95-positive glutamatergic synapses on the dendrites of cortical neurons, which can be blocked by the NMDAR antagonist 7-CK (Figures 10A'–L'). Quantification confirms that D-serine, but not glycine, significantly increases the number of glutamatergic synapses on the dendrites, and that this increase can be blocked by 7-CK (Figure 10M). The findings thus demonstrate that D-serine, unlike glycine, stabilizes glutamatergic synapses on the dendrites of cortical neurons through NMDARs.

## Discussion

The present study uses *in vitro* cultured cortical neurons to show that D-serine and SR are associated with PSD-95 and NMDARs in postsynaptic neurons and with glutamatergic synapse stability. Endogenous D-serine and SR colocalize with PSD-95, but not presynaptic VGLUT1, and is highly enriched in the postsynaptic density of glutamatergic synapses of cortical neurons. Low-density astrocytes in cortical cultures lack SR expression but contain enriched D-serine in large vesicle-like structures, suggesting possible synthesis of D-serine in postsynaptic neurons and separate storage of D-serine in astrocytes. More interestingly, endogenous D-serine and SR colocalize with PSD-95 in the postsynaptic terminals of glutamatergic synapses during early and late synaptic development, implicating D-serine/SR involvement in glutamatergic synaptic development. Exogenous application of D-serine enhances SR interactions with PSD-95 and NMDARs in a postsynaptic protein complex and increases the number of glutamatergic synapses, suggesting that exogenous D-serine enhances postsynaptic SR/PSD-95 signaling and stabilizes glutamatergic synapses during synaptic development. This is blocked by the NMDAR antagonists AP5 and 7-CK, a specific antagonist at the glycine site of NMDARs. In contrast, exogenous application of glycine has no such effects, suggesting that the D-serine effects are mediated through synaptic NMDARs. The present results thus implicate D-serine and SR in control of PSD-95 signaling and glutamatergic synapse stability during cortical synaptic and circuit development.

The localization of SR and D-serine in astrocytes and neurons remains disputed. SR was initially localized to astrocytes, but later found prominently in neurons (Schell et al., 1997; Wolosker et al., 1999, 2008; Kartvelishvily et al., 2006; Martineau et al., 2006, 2014; Panatier et al., 2006; Miya et al., 2008; Benneyworth et al., 2012; Ehmsen et al., 2013; Balu et al., 2014; Mothet et al., 2015). Our results show that SR is a postsynaptic protein that colocalizes with PSD-95, a glutamatergic postsynaptic terminal marker, but not presynaptic marker VGLUT1, in the glutamatergic synapses of cortical glutamatergic and GABAergic neurons. Co-immunoprecipitation confirms endogenous SR interactions with PSD-95 and NMDARs postsynaptically and co-immunostaining shows that D-serine appears enriched in PSD-95-positive glutamatergic postsynaptic terminals on cortical neurons, suggesting D-serine may be synthesized and produced in the postsynaptic terminals. Low-density astrocytes in primary cortical neuronal cultures lack endogenous SR expression but contain enriched D-serine in large vesicle-like structures (1–3 μm), suggesting possible storage of D-serine in astrocytes. This matches the findings from other labs that D-serine is localized in astrocytic synaptic-like vesicles and released as large vesicles (1–3 μm) in astrocytes (Kang et al., 2013; Martineau et al., 2013). The presence of the D-serine degrading enzyme DAAO in astrocytes further suggests possible degradation of D-serine in astrocytes. Our findings thus implicate both neuronal and astrocytic D-serine in synaptic development and postsynaptic functions.

Interactions between SR and PSD-95 are involved in coupling synaptic NMDAR and AMPAR activities and functions (Ma et al., 2014). We found that exogenous D-serine application modulates postsynaptic SR/PSD-95/NMDAR interactions in cortical neurons, implicating D-serine in SR/PSD-95 signaling and postsynaptic functions. SR is a highly regulated enzyme that interacts with several NMDAR- and AMPA receptor (AMPAR)-interacting proteins including GRIP1, PICK1, DISC1, stargazin, and PSD-95 (Kim et al., 2005b; Fujii et al., 2006; Hikida et al., 2008; Ma et al., 2013, 2014). The physiological interactions of SR with these receptor-interacting proteins may affect SR translocation to the plasma membrane and SR catalytic activity as well as synaptic NMDAR-AMPAR activities. For instance, NMDAR activation promotes translocation of SR to the plasma membrane, which dramatically reduces the enzyme activity (Balan et al., 2009). AMPAR activation dissociates SR from the protein complex on the membranes and translocates it to the cytosol leading to enhanced activity of SR to generate more D-serine (Ma et al., 2014). SR interactions with DISC1, PICK1, and GRIP1 also modulate SR activity and D-serine production (Kim et al., 2005b; Ma et al., 2014; Nomura et al., 2016). Our findings suggest that D-serine may control postsynaptic SR activity and D-serine production through modulation of SR interactions with PSD-95, NMDAR and other postsynaptic scaffold proteins. Furthermore, PSD-95 is a crucial postsynaptic scaffold protein that regulates glutamatergic synapse formation and maturation (Migaud et al., 1998; El-Husseini et al., 2000; Nikonenko et al., 2008). For example, PSD-95 promotes spine synapse formation through direct interaction with postsynaptic neuronal nitric oxide synthase (nNOS) and drives presynaptic and postsynaptic maturation of glutamatergic synapses (El-Husseini et al., 2000; Nikonenko et al., 2008). PSD-95 also controls synaptic AMPAR number through direct interaction with stargazin (Schnell et al., 2002). Therefore, our findings suggest that D-serine could modulate postsynaptic PSD-95 signaling, possibly through interactions with nNOS and synaptic AMPARs, thereby modulating glutamatergic synaptic development.

Our findings further reveal that endogenous D-serine and SR colocalize with PSD-95 in the postsynaptic terminals of glutamatergic synapses during early and late synaptic development. Exogenous D-serine indeed increases the number of VGLUT1- and PSD95-positive glutamatergic synapses on the dendrites of cortical neurons, implicating D-serine in control of glutamatergic synapse stability during cortical synaptic and circuit development. Our findings also show that the D-serine effects on glutamatergic synapses appears more pronounced on presynaptic than postsynaptic terminals, suggesting that D-serine may stabilize glutamatergic synapses through nitric oxide retrograde signaling via postsynaptic PSD-95/NOS interactions. SR *null* mutant mice, which have less than 10% of normal brain D-serine and a schizophrenia-like phenotype, have reduced dendritic spine density that can be partially rescued by chronic D-serine treatment (Balu and Coyle, 2012; Balu et al., 2012, 2014). In addition, disruption of D-serine synthesis in mice during early postnatal life leads to schizophrenia-like behavioral abnormalities in adulthood, which can be rescued by chronic D-serine treatment during juvenile life (Hagiwara et al., 2013). Similarly, targeted deletion of the SR-interacting protein PICK1 in mice leads to D-serine deficiency in prefrontal cortex and schizophrenia-like behavioral abnormalities. The abnormalities can be rescued by transient neonatal supplementation of D-serine, but not by a similar treatment in adulthood, implicating D-serine in brain development (Nomura et al., 2016). Our findings suggest that D-serine/SR may regulate cortical glutamatergic synaptic development, which could be disrupted by abnormal D-serine/SR levels in early life leading to permanent deficits in adulthood in schizophrenia. Hence, D-serine treatment may provide a potential therapeutic for rescuing synaptic deficits in schizophrenia.

Synaptic and extrasynaptic NMDARs are associated with differential gene expression and have different roles in synaptic plasticity and cell death (Hardingham and Bading, 2010; Gladding and Raymond, 2011; Kaufman et al., 2012; Papouin et al., 2012; Karpova et al., 2013; Parsons and Raymond, 2014). D-serine preferentially gates synaptic NMDARs while glycine preferentially gates extrasynaptic NMDARs (Papouin et al., 2012). Our studies show that D-serine and glycine have differential effects on postsynaptic SR/PSD-95 signaling and glutamatergic synapse stability, implicating D-serine, rather than glycine, in controlling postsynaptic protein signaling and glutamatergic synaptic development. This is likely mediated by synaptic, rather than extrasynaptic, NMDARs. Synaptic NMDARs play crucial roles in many forms of synaptic plasticity, such as LTP and LTD. Similarly, the selective control of synaptic NMDARs may provide a mechanism by which D-serine regulates glutamatergic synaptic development and function.

## Author contributions

HL designed and performed the experiments, analyzed and interpreted the data, and wrote the paper. AJ performed the experiments and analyzed the data. SA wrote the paper. DL designed the experiments, interpreted the data and wrote the paper.

### Conflict of interest statement

The authors declare that the research was conducted in the absence of any commercial or financial relationships that could be construed as a potential conflict of interest. The reviewer NS and handling Editor declared their shared affiliation, and the handling Editor states that the process nevertheless met the standards of a fair and objective review.
