# Supplementary material for: D-Serine and Serine Racemase Are Associated with PSD-95 and Glutamatergic Synapse Stability
Source: Front Cell Neurosci. 2016 Feb 25;10:34. doi: 10.3389/fncel.2016.00034 (PMC4766304; doi:10.3389/fncel.2016.00034)
Supplement: Supplementary file 1 [file DataSheet1.PDF]

*Supplementary Material*

**D-serine and serine racemase are associated with PSD-95 and glutamatergic synapse stability**

Hong Lin[1], Ariel A. Jacobi[1,2] , Stewart A. Anderson[3,4] and David R. Lynch[1,4*]

*** Correspondence**: David R. Lynch, MD, PhD, lynchd@mail.med.upenn.edu

## Supplemental Figure Legends

**Supplemental Figure 1 Characterization of cortical glutamatergic and GABAergic neurons in cultures.** **A-F**, Confocal images of GABA (**A** and **D**) immunofluorescence (red) and PSD95 (**B** and **E**) immunofluorescence (green) showing that, in cortical GABAergic interneurons (grey arrows), PSD95 is localized in the shaft-like synapses on the somatic and dendritic membrane while GABA is distributed in the soma, dendrites as well as axonal and presynaptic terminals surrounding glutamatergic neurons (white arrows). **G-I**, Confocal images of GABA (**G**) immunofluorescence (red) and PSD95 (**H**) immunofluorescence (green) showing that PSD95 is distributed in the soma and in the spine-like synapses on the dendrites of cortical glutamatergic neurons surrounded by GABA-positive presynaptic terminals. **J-O**, Confocal images of VGLUT1 (**J** and **M**) immunofluorescence (red) and PSD95 (**K** and **N**) immunofluorescence (green) further confirming VGLUT1- and PSD95-positive shaft-like synapses on cortical GABAergic interneurons (**J-L**) as well as dendritic spine-like synapses on cortical glutamatergic neurons (**M-O**) in cortical cultures. Scale bars as indicated.

**Supplemental Figure 2 SR is absent in GABAergic presynaptic and postsynaptic terminals on cortical glutamatergic and GABAergic neurons.** Confocal images (**A-F**) of SR (**A** and **D**)

immunofluorescence (green) and GAD65 (**D** and **E**) immunofluorescence (red) shows that SR does not colocalize with GAD65-positive GABAergic presynaptic terminals on cortical glutamatergic (**C**) and GABAergic (**F**) neurons which are characterized by SR and PSD95 immunofluorescence shown in Fig. 1 and Supplemental Fig. 1. Confocal images (**G-L)** of SR (**G** and **J**) immunofluorescence (red) and gephyrin (**H** and **K**) immunofluorescence (green) shows that SR does not colocalize with gephyrin-positive GABAergic postsynaptic terminals on cortical glutamatergic (**I**) and GABAergic (**L)** neurons Scale bars as indicated.

**Supplemental Figure 3 The specificity of α-D-serine immunoreactivities in neurons and astrocytes.** Confocal images of D-serine (**A**) and L-serine (**E**) immunofluorescence (red), GFAP (**B** and **F**) immunofluorescence (green) and MAP2 (**C** and **G)** immunofluorescence (blue) showing that D-serine appears as puncta abundantly in the soma and dendrites of MAP2-positive cortical neurons and enriched in the soma of astrocytes (**D**), whereas L-serine appears diffusely distributed in GFAP-positive astrocytes and very low in cortical neurons (**H**), suggesting the specificities of α-D-serine immunoreactivities. Scale bars as indicated.
