# Supplementary figures and images for: D-Serine and Serine Racemase Are Associated with PSD-95 and Glutamatergic Synapse Stability
Source: Front Cell Neurosci. 2016 Feb 25;10:34. doi: 10.3389/fncel.2016.00034 (PMC4766304; doi:10.3389/fncel.2016.00034)

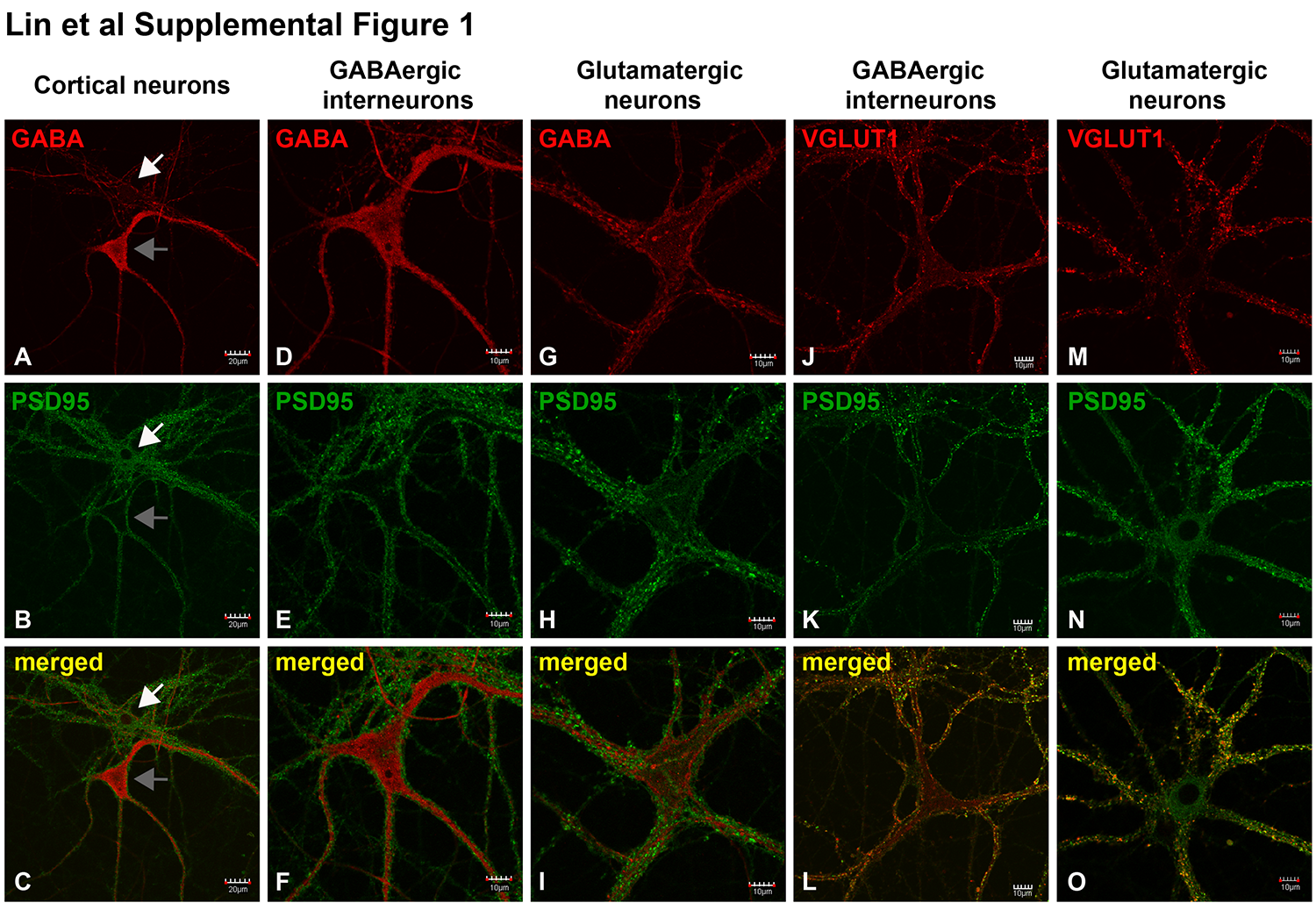

Supplement: Supplementary file 2 [file Image1.TIF]

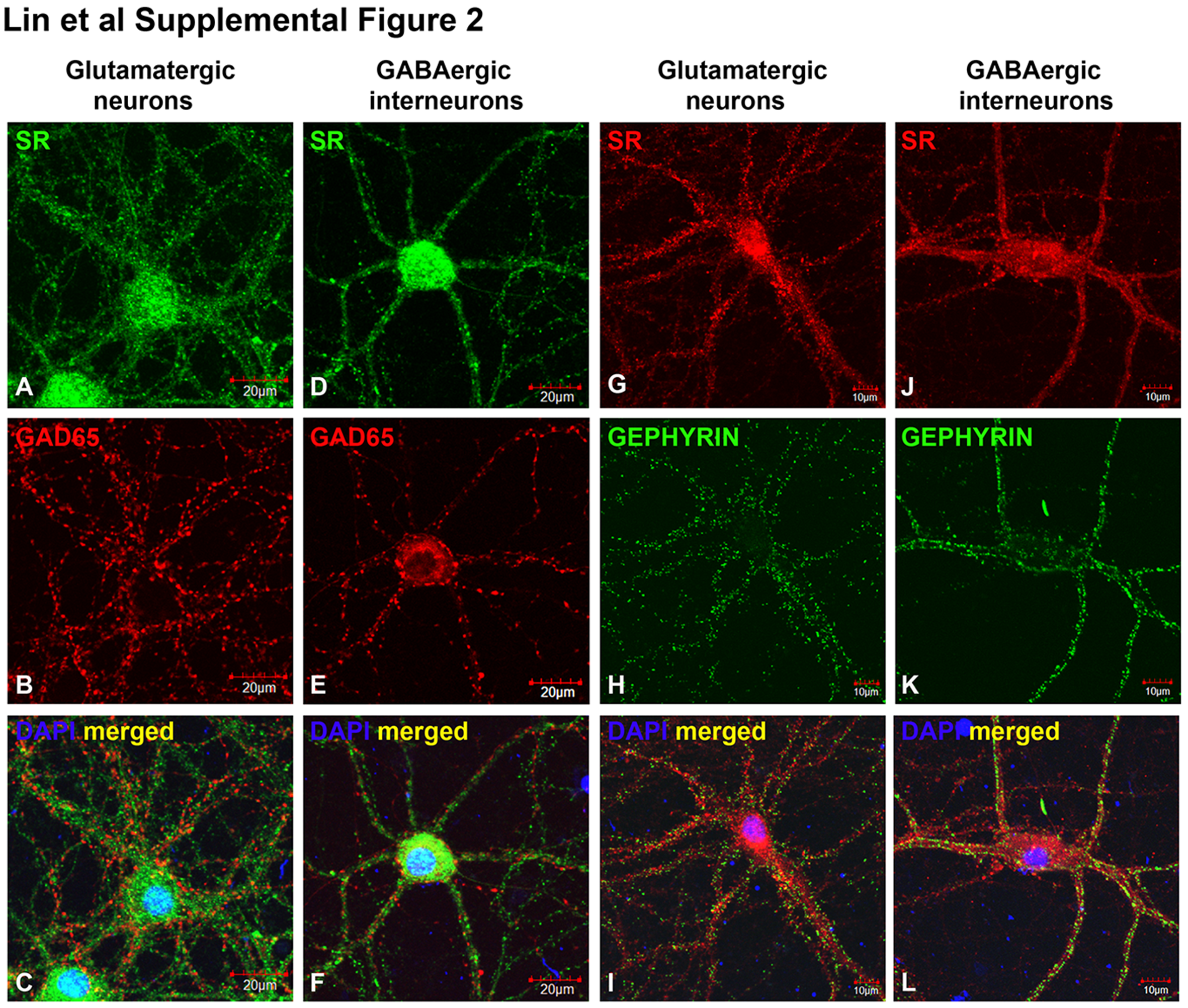

Supplement: Supplementary file 3 [file Image2.TIF]

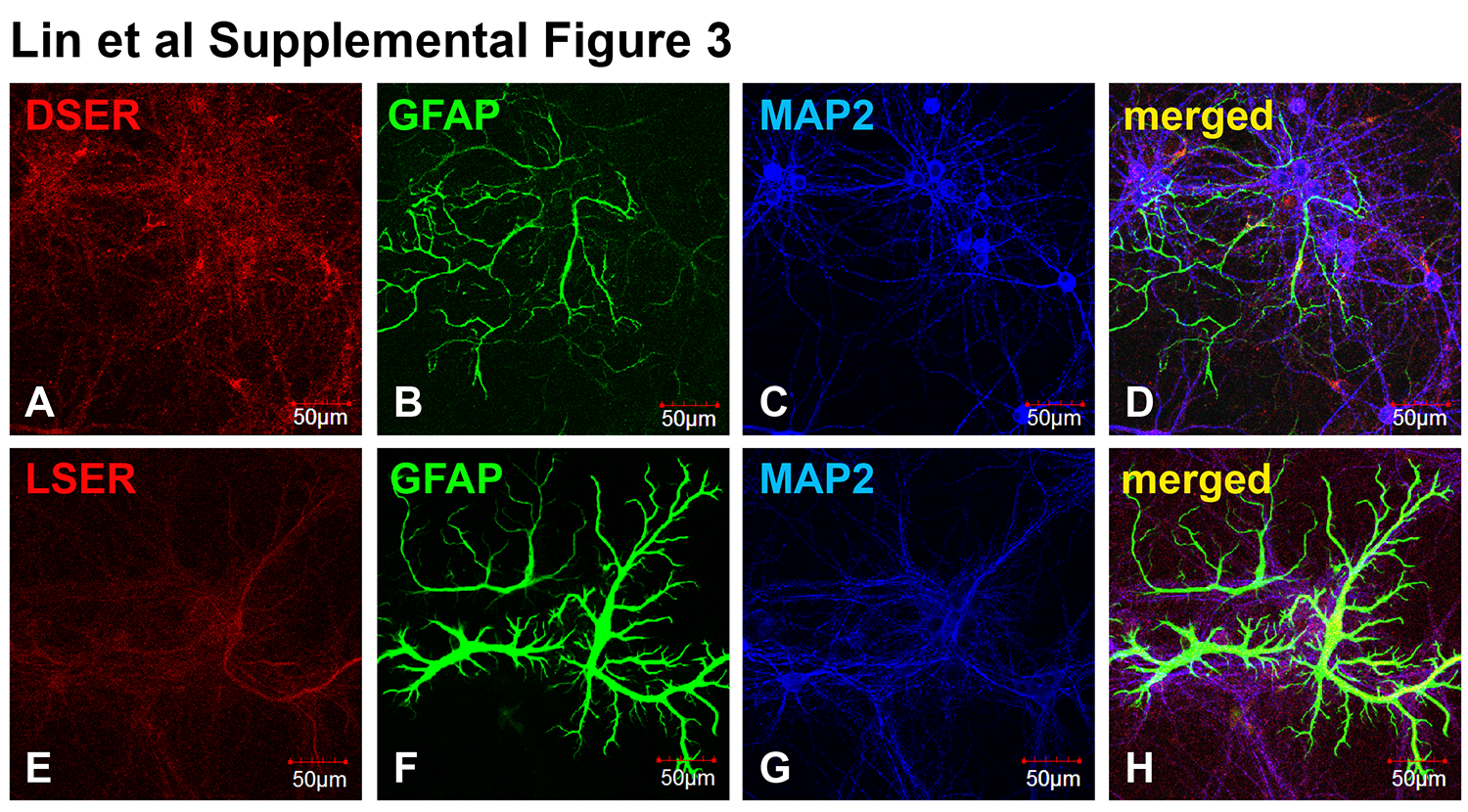

Supplement: Supplementary file 4 [file Image3.TIF]
